# Local administration of mRNA encoding cytokine cocktail confers potent anti-tumor immunity

**DOI:** 10.3389/fimmu.2024.1455019

**Published:** 2024-09-03

**Authors:** Zhigang Li, Ling Hu, Yi Wang, Qi Liu, Jun Liu, Haiyan Long, Qi Li, Liping Luo, Yucai Peng

**Affiliations:** Department of Research and Development, Liverna Therapeutics Inc., Zhuhai, China

**Keywords:** cytokine, mRNA, cancer immunotherapy, intratumoral administration, interleukin-12

## Abstract

Immunotherapy using inflammatory cytokines, such as interleukin (IL)-2 and interferon (IFN)-α, has been clinically validated in treating various cancers. However, systemic immunocytokine-based therapies are limited by the short half-life of recombinant proteins and severe dose-limiting toxicities. In this study, we exploited local immunotherapy by intratumoral administration of lipid nanoparticle (LNP)-encapsulated mRNA cocktail encoding cytokines IL-12, IL-7, and IFN-α. The cytokine mRNA cocktail induced tumor regression in multiple syngeneic mouse models and anti-tumor immune memory in one syngeneic mouse model. Additionally, immune checkpoint blockade further enhanced the anti-tumor efficacy of the cytokine mRNAs. Furthermore, human cytokine mRNAs exhibited robust anti-tumor efficacy in humanized mouse tumor models. Mechanistically, cytokine mRNAs induced tumor microenvironment inflammation, characterized by robust T cell infiltration and significant inflammatory cytokine and chemokine production.

## Introduction

Cancer immunotherapy eliminates tumor cells by boosting host immunity. It has remarkably altered the paradigm of cancer therapy and significantly improved the clinical outcomes of cancer patients. Immune checkpoint blockade (ICB) with anti-programmed cell death protein 1 (PD-1)/programmed cell death-ligand 1 (PD-L1) and anti-cytotoxic T-lymphocyte associated protein 4 monoclonal antibodies confers durable clinical responses and prolongs survival of patients with various solid tumors (1). However, the overall response rate of immune checkpoint inhibitors (ICIs) is relatively low, with many tumors being resistant to ICIs (2). Combination therapy with ICIs and chemotherapy, radiotherapy, or targeted therapy has shown synergistic clinical response and improved clinical benefits in several metastatic malignancies (3).

Inflammatory cytokines play an important role in regulating innate and adaptive immune responses, and some cytokines have been found to exhibit potent anti-tumor activity in multiple animal models as well as clinical studies (4, 5). Among these cytokines, the mechanism of action and clinical outcomes of interleukin (IL)-12 are well characterized (6–9). IL-12 is a central mediator of the Th1 immune response, and it enhances the activation and cytotoxicity of CD8^+^ T cells, natural killer (NK) cells, and NK T (NKT) cells (10–12). IL-12 is also associated with polarizing macrophage from the anti-inflammatory M2 state to the pro-inflammatory M1 state (13, 14). Cytokine IL-7 is essential for T cell homeostasis and promotes memory T cell survival and expansion (15). IL-12 and IL-7 synergistically induce T cell activation and NK cell maturation, leading to potent anti-tumor activity (16–18). Type I interferons (IFNs), such as IFN-α, exert anti-cancer effects by directly inducing tumor cell apoptosis (19–21). Additionally, IFN-α stimulates dendritic cell maturation and enhances antigen processing and presentation (22, 23). Moreover, several clinical trials showed the promising therapeutic effects of IL-12, IL-7, and IFN-α in cancer treatment (24–26), with IFN-α specifically being approved for the treatment of melanoma (27).

Systemic administration of recombinant IL-12 confers promising clinical anti-cancer efficacy; however, it is limited by its poor tolerability. Intratumoral administration of IL-12-encoding adenovirus, oncolytic virus, or plasmid DNA (tavokinogene telseplasmid) has been found to upregulate local IL-12 production and lead to anti-tumor immune responses in preclinical mouse models and clinical trials (28–32). However, these approaches may induce deleterious genomic rearrangements and anti-viral vector immunity. In contrast, local mRNA therapy could ensure transient and local translation of cytokines without using viral vectors or causing gene integration in host genomic DNA. Furthermore, early clinical studies revealed that intratumoral administration of cytokine mRNAs, such as MEDI1191 (encoding human IL-12) (33), mRNA-2752 (encoding human OX40L, IL-23, and IL-36γ) (34), and SAR441000 (encoding human IL-12, IFN-α2b, GM-CSF, and IL-15) (35), is well-tolerated.

In this study, we generated lipid nanoparticle (LNP)-encapsulated cytokine mRNAs encoding IL-12, IL-7, and IFN-α as anti-tumor drug candidates and evaluated the therapeutic benefits of intratumoral administration of IL-12/IL-7/IFN-α triplet. In most of the tested murine tumor models, the LNP-encapsulated mRNA cocktail induced significant tumor shrinkage and prolonged animal survival. Additionally, combination therapy with cytokine mRNAs and ICB synergistically induced potent anti-tumor efficacy. Mechanistically, IL-12/IL-7/IFN-α mRNA triplet altered the TME by boosting inflammatory immune responses, as illustrated by a significant increase in T cell infiltration and cytokine and chemokine production.

## Materials and methods

### Cytokine mRNA formulation

A single-chain IL-12 was constructed by linking the amino acid sequences of IL-12p40 (Uniprot mouse P43432 or human P29460) and IL-12p35 (Uniprot mouse P43431 or human P29459) by a peptide linker (GSSGGGGSPGGGSS). The signal peptide of IL-12p40 was retained for secretion, while that of IL-12p35 was removed. Similarly, IL-7 and IFN-α were constructed using their corresponding wild-type amino acid sequences, namely Uniprot mouse IL-7 P10168, Uniprot human IL-7 P13232, Uniprot mouse IFN-α P07351, and Uniprot human IFN-α P01563. The protein sequences were back-translated, and the DNA sequences were optimized for higher protein expression based on in-silico analysis using algorithm of “Population Immune Algorithm”, which takes advantage of both population genetics and immunology theories. The optimized open reading frame sequences flanked by 5’UTR, 3’UTR, and poly-A sequences were then integrated into plasmid vectors. Finally, the cytokine mRNAs were produced and encapsulated by LNP using the Liverna Therapeutics platform (China patent ZL201911042634.2), as described previously (36–38). Briefly, mRNAs were produced by *in vitro* transcription (IVT), and purified using Oligo-dT affinity column (Sepax Technologies, Inc., China) and Tangential Flow Filtration (TFF, Repligen Corporation, America). Purified mRNAs were than encapsulated in LNPs. Briefly, the MC3 ionizable lipid, DSPC, cholesterol and DMG-PEG2000 were dissolved in ethanol and rapidly mixed with citrate buffer using a microfluidic machine (Precision Nanosystem, Inc). The analytical characterization of the product was conducted, including the determination of particle size, polydispersity, encapsulation, endotoxin levels, and bioburden. The mRNA concentration in the product was determined by UV spectrometer (TU-1810PC, Persee).

### Syngeneic tumor model and humanized mouse model

The Balb/c mice and C57BL/6 mice (female, 6–8 weeks old, 18–23 g) were purchased from GemPharmatech Co., Ltd. (Nanjing, China), Cyagen Co., Ltd. (Guangzhou, China), and Zhuhai BesTest BioTech Co., Ltd. (Zhuhai, China). The huHSC-NCG-hIL-15 mice (huCD34^+^HSC-NOD/ShiLtJGpt-Prkdc^em26Cd52^ Il2rg^em26Cd22^ Il15^em1Cin(hIL15)^/Gpt(CH), strain no. T038070) and NCG-MHC-dKO mice (NOD/ShiLtJGpt-Prkdc^em26Cd52^ Il2rg^em26Cd22^ H2K1^em2Cd10^ H2D1^em2Cd6in16de11in5^ H2Ab1^em2Cd1^/Gpt, strain no. T050886) were purchased from GemPharmatech Co., Ltd. (China).

Syngeneic colorectal tumor, melanoma, and breast cancer models were established by subcutaneously injecting CT26 cells (1×10^6^ in 100 μl of Dulbecco’s phosphate-buffered saline, DPBS), B16F10 cells (1×10^6^ in 100 μl DPBS), and 4T-1 cells (3×10^5^ in 100 μl of DPBS) at the right flank of Balb/c mice, C57BL/6 mice, and Balb/c mice, respectively.

The huHSC-NCG-hIL15 model was established by engrafting NCG-hIL-15 mice with human hematopoietic stem cells (huHSCs). After 11–14 weeks, the reconstituted human immune cells (T cells, B cells, and NK cells) were confirmed by flow cytometry analysis of mouse peripheral blood samples. The mice with 20–80% human CD45^+^ immune cells were selected and subcutaneously xenografted with human breast cancer MDA-MB-231 cells (5×10^6^ cells in 150 μl of DPBS with 50% Matrigel). The huPBMC-NCG-MHC-dKO model was established by intraperitoneally engrafting NCG-MHC-dKO mice with human peripheral blood mononuclear cells (PBMCs, 1×10^7^ cells in 200 μl of DPBS), followed by MDA-MB-231 cells after 24 h, as described above.

The width (smaller diameter) and the length (larger diameter) of the tumors was measured 2–3 times a week by a caliper. The volumes of the tumors were calculated using the following formula: tumor volume = (length × width^2^)/2. The mice were randomly grouped for *in vivo* drug administration when the average tumor volume was approximately 80–100 mm^3^. The LNP-encapsulated cytokine mRNAs were intratumorally injected in a fixed volume (50 μl) at the indicated dose once a week for 3 consecutive weeks, unless stated otherwise. mRNA encoding firefly luciferase was used as a negative control in most of the experiments, except for the one indicated in figure legend, where phosphate-buffered saline served as a negative control. For ICI treatment, anti-mouse PD-1 antibodies (Cat. #BE0146, BioXCell) or anti-human PD-L1 antibodies (Tecentriq, Roche, Switzerland) were intraperitoneally injected at a dose of 10 mg/kg twice a week for 3 consecutive weeks. The day of the first dose treatment was assigned as day 0. Mice were checked daily for adverse clinical reactions. The tumor size and body weight of individual mice were measured 2–3 times a week. Survival events were recorded upon death or euthanasia due to exceeded tumor volume or adverse effects.

### *In vivo* bioimaging

The LNP-encapsulated mRNAs encoding the firefly luciferase gene were intratumorally injected into MDA-MB-231 tumor-bearing huHSC-NCG-hIL15 mice at a dose of 12 μg/mouse once a week for 3 consecutive weeks. After 6 and 24 h post each injection, the luciferase signals were analyzed by *in vivo* bioluminescence imaging using the Xenogen IVIS Spectrum Imaging System (Caliper Life Sciences, Hopkinton, MA, USA). After 10 min, L-luciferin was intraperitoneally injected and the luciferase activity was quantified in live animals, with an exposure time of 1 min. The regions of interest were quantified as average radiance (photons/[s cm^2^ sr]) represented as color-scaled images superimposed on grayscale photos of mice using Living Image software (Caliper Life Sciences).

### Demonstration of cytokine expression

To explore *in vitro* cytokine expression, 5 μg of LNP-encapsulated mRNAs encoding mouse or human IL-12, IL-7, or IFN-α was transfected individually to Jurkat cells (1×10^6^ cells, American Type Culture Collection, Manassas, VA, USA) cultured in RPMI 1640 medium. LNP-encapsulated luciferase mRNA was used as a negative control. After 24 h, the culture medium was collected and the cytokine levels were quantified by enzyme-linked immunosorbent assay (ELISA).

To explore *in vivo* cytokine expression, the CT26 tumor-bearing Balb/c mice (with ~100 mm^3^ tumor volume) were intratumorally injected with 30 μg of mouse IL-12/IL-7/IFN-α mRNA triplet (10 μg of each). LNP-encapsulated luciferase mRNA was used as a negative control. After 6 h, the peripheral blood and tumor tissues (homogenized) were collected for ELISA.

QuantiCyto^®^ mouse IL-12 (Cat. #EMC006), mouse IFN-α (Cat. #EMC035a), human IL-12 (Cat. #EHC010QT), human IL-7 (Cat. #EHC149), and human IFN-α (Cat. #EHC030a) ELISA kits were purchased from NeoBioscience Technology Co., Ltd (Shenzhen, China). Mouse IL-7 ELISA kit (Cat. #EK207) was purchased from Multi Sciences (Lianke) Biotech Co., Ltd. (Hangzhou, China).

### Bioactivity analysis

To demonstrate the bioactivities of IL-12, IL-7, and IFN-α, Jurkat cells were first transfected with LNP-encapsulated human IL-12, IL-7, or IFN-α mRNAs. LNP-encapsulated luciferase mRNA was used as a negative control. Thereafter, their culture supernatants were collected after 24 h and used to stimulate NK92 cells, human PBMCs, and human monocytic cell line THP-1 (American Type Culture Collection), respectively. After 24 h of stimulation, the NK92 cells, human PBMCs, and THP-1 cells were stained with Alexa Fluor 647-conjugated anti-STAT4 (pY693, Cat. #558137, BD biosciences, Franklin Lakes, NJ, USA), phycoerythrin-conjugated anti-STAT5 (pY694, Cat. #612567, BD biosciences), and Alexa Fluor 647-conjugated anti-STAT1 (Tyr701, Cat. #666410, BioLegend, San Diego, CA, USA) antibodies, respectively, and analyzed by flow cytometry, following the manufacturer’s instructions.

### Serum cytokine measurement

The MDA-MB-231 tumor-bearing huHSC-NCG-hIL-15 mice were intratumorally treated with a single dose of 5 μg human cytokine mRNA triplet (3:1:1 of IL-12/IL-7/IFN-α). LNP-encapsulated luciferase mRNA was used as a negative control. Subsequently, their peripheral blood samples were collected for cytometric bead array analysis (Cat. #558264, BD Biosciences) of serum human IFN-γ (Cat. #558269, BD Biosciences) and human IFN-γ-induced protein 10 kDa (IP-10, Cat. #558280, BD Biosciences) levels, following the manufacturer’s instructions.

### Flow cytometry analysis

The CT26 tumor-bearing mice were intratumorally injected with mouse cytokine IL-12/IL-7/IFN-α mRNA triplet at a dose of 30 μg/week (10 μg of each) for 2 weeks, and the tumors were collected at day 11 after the first treatment. LNP-encapsulated luciferase mRNA was used as a negative control. The tumor samples were dissociated into single cells and blocked with TruStain FcX (anti-mouse CD16/32, Cat. #101320, BioLegend). Subsequently, the samples were stained with viability dye (Cat. #S10274, Invitrogen, Waltham, MA, USA) and the following fluorescent antibodies: anti-mouse CD45 (Cat. #563891, BioLegend), anti-mouse CD3 (Cat. #100349, BioLegend), anti-mouse CD4 (Cat. #560181, BD Biosciences), anti-mouse CD8 (Cat. #553033, BD Biosciences), anti-mouse NKp46 (Cat. #560755, BioLegend), anti-mouse CD11b (Cat. #562287, BD Biosciences), anti-mouse Ly6G (Cat. #127628, BioLegend); anti-mouse Ly6C (Cat. #553104, BD Biosciences), and anti-mouse F4/80 (Cat. #63-4801-82, eBioscience, San Diego, CA, USA). The samples were assessed using the Attune™ NxT Flow Cytometer (Thermo Fisher, Waltham, MA, USA), and the data were analyzed using Flowjo v10.8 (BD Biosciences).

Flow cytometry gating strategies included singlet gates (FSC-A/FSC-H) and live cells (low or negative with viability dye staining) to identify immune cell phenotypes, as follows: mouse leukocytes: mCD45^+^; mouse T cells: mCD45^+^, mCD11b^−^, mNKp46^−^, and mCD3^+^; mouse CD4^+^ T cells: mCD45^+^, mCD11b^−^, mNKp46^−^, mCD3^+^, mCD8^−^, and mCD4^+^; mouse CD8^+^ T cells: mCD45^+^, mCD11b^−^, mNKp46^−^, mCD3^+^, mCD4^−^, and mCD8^+^; mouse NK cells: mCD45^+^, mCD11b^−^, mCD3^−^, and mNKp46^+^; mouse macrophages: mCD45^+^, mCD11b^+^, mLy6C^−^, mLy6G^−^, and mF4/80^+^; mouse granulocytic myeloid-derived suppressor cells: mCD45^+^, mCD11b^+^, mLy6G^+^, and mLy6C^+^; and mouse monocytic myeloid-derived suppressor cells: mCD45^+^, mCD11b^+^, mLy6G^−^, and mLy6C^+^.

### Statistical analysis

Statistical analyses were performed using GraphPad Prism 8 software (La Jolla, CA, USA). Data are presented as the mean ± standard error of the mean or the mean ± standard deviation, as specified. Statistical analysis was conducted with unpaired two-tailed Student’s *t*-tests, two-way ANOVA with Sidak’s multiple comparison test, or Log-rank (Mantel-Cox) tests, as indicated in the figure legends. An adjusted *P* value of ≤ 0.05 was considered indicative of a statistically significant difference. Statistical differences are denoted as *P ≤ 0.05, **P ≤ 0.01, and ***P ≤ 0.001.

## Results

### Demonstration of protein translation from mRNAs

**Figure 1:**
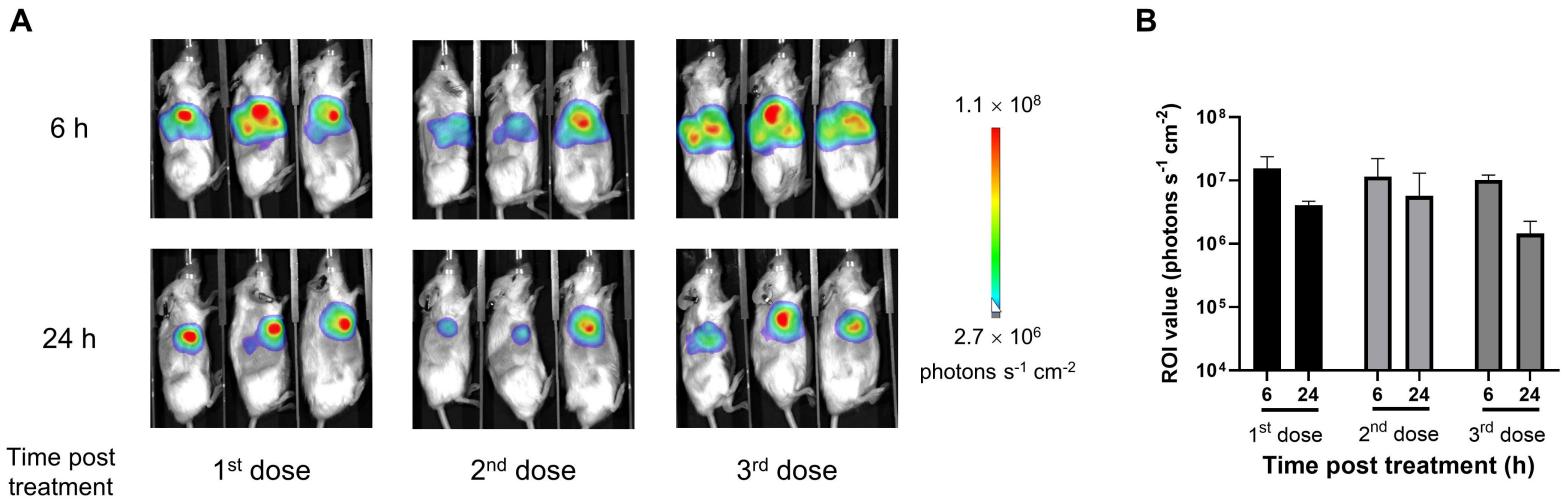
Intratumoral administration of lipid nanoparticle (LNP)-encapsulated mRNA increased local protein expression in tumor tissues. The subcutaneous MDA-MB-231 tumor-bearing huHSC-NCG-hIL15 mice were intratumorally injected with 12 μg of LNP-encapsulated luciferase mRNA once a week for 3 consecutive weeks. **(A)** *In vivo* bioluminescence images were obtained 6 and 24 h post each injection. **(B)** Luciferase signals analyzed by *in vivo* bioluminescence imaging and quantification of regions of interest (ROIs). Data are presented as the mean ± standard deviation with n = 3.

The *in vivo* translation of local mRNA delivery was demonstrated by intratumorally injecting LNP-encapsulated mRNAs encoding firefly luciferase into tumor-bearing mice and monitoring their bioluminescence activities, which reflect the exogenous gene expression. Strong luciferase signals were observed locally within the tumor after 6 h post each injection, which lasted for at least 24 h (**Figure 1**). No obvious bioluminescence was detected distally in other tissues. These results confirm the protein expression by intratumoral mRNA administration.

### Demonstration of *in vitro* and *in vivo* cytokine mRNA expression

**Figure 2:**
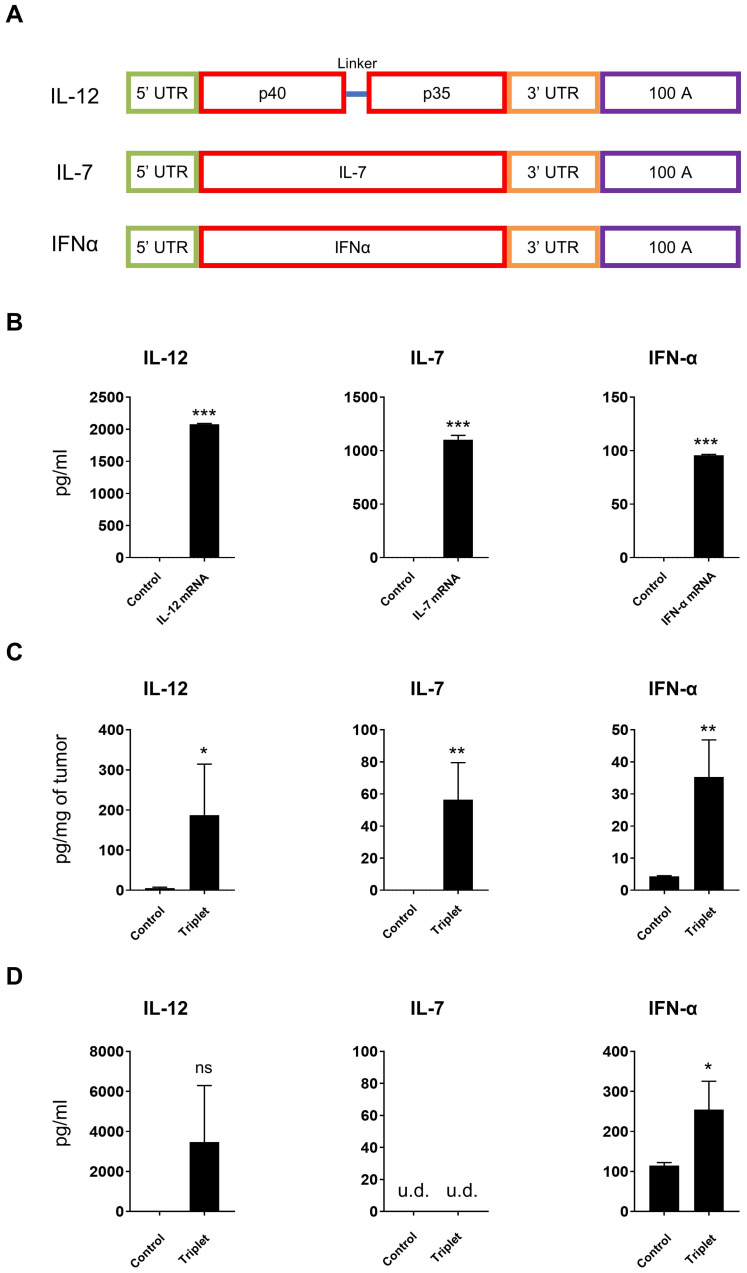
Cytokine mRNAs expressed *in vitro* and *in vivo*. **(A)** Schematic diagram of cytokine mRNA construction. **(B)** Cytokines secreted from transfected Jurkat cells were measured by ELISA. **(C, D)** CT26 tumor-bearing mice were intratumorally injected with 30 μg of cytokine mRNA triplet (10 μg of each) and the corresponding cytokine levels were detected in the tumor tissues **(C)** and serum samples **(D)** by ELISA. Luciferase mRNA was used as a negative control. u.d., undetectable (below the detection limit of the assay). Data are presented as the mean ± standard deviation, with n = 3. Statistical analysis was conducted using an unpaired, two-tailed Student’s *t*-test; ns, not significant, *P ≤ 0.05, **P ≤ 0.01, ***P ≤ 0.001. Data in **(B)** are representative of three independent experiments.

Since our results demonstrated the feasibility of intratumoral delivery of LNP-encapsulated mRNAs, we subsequently explored the therapeutic potential of IL-12/IL-7/IFN-α mRNA triplet. We first generated a mouse cytokine mRNA triplet (**Figure 2A**), as human IL-12 and IFN-α are not bioactive in mice. The cytokine mRNAs encoding IL-12, IL-7, or IFN-α were individually transfected into Jurkat cells, and the expression of the corresponding cytokines was observed by ELISA. All three cytokines were well expressed and secreted in the Jurkat cell culture supernatant (**Figure 2B**).

The subcutaneous CT26 tumor-bearing syngeneic mice model was used to explore the *in vivo* expression of LNP-encapsulated mRNAs. Intratumoral injection of LNP-encapsulated IL-12/IL-7/IFN-α mRNA triplet significantly increased cytokine expression in the tumor tissues (**Figure 2C**). Additionally, IL-12 and IFN-α levels were increased in the serum samples (**Figure 2D**), which is likely due to the secretion of exogenous cytokines in the periphery.

### Cytokine mRNAs induced tumor regression in syngeneic murine models

**Figure 3:**
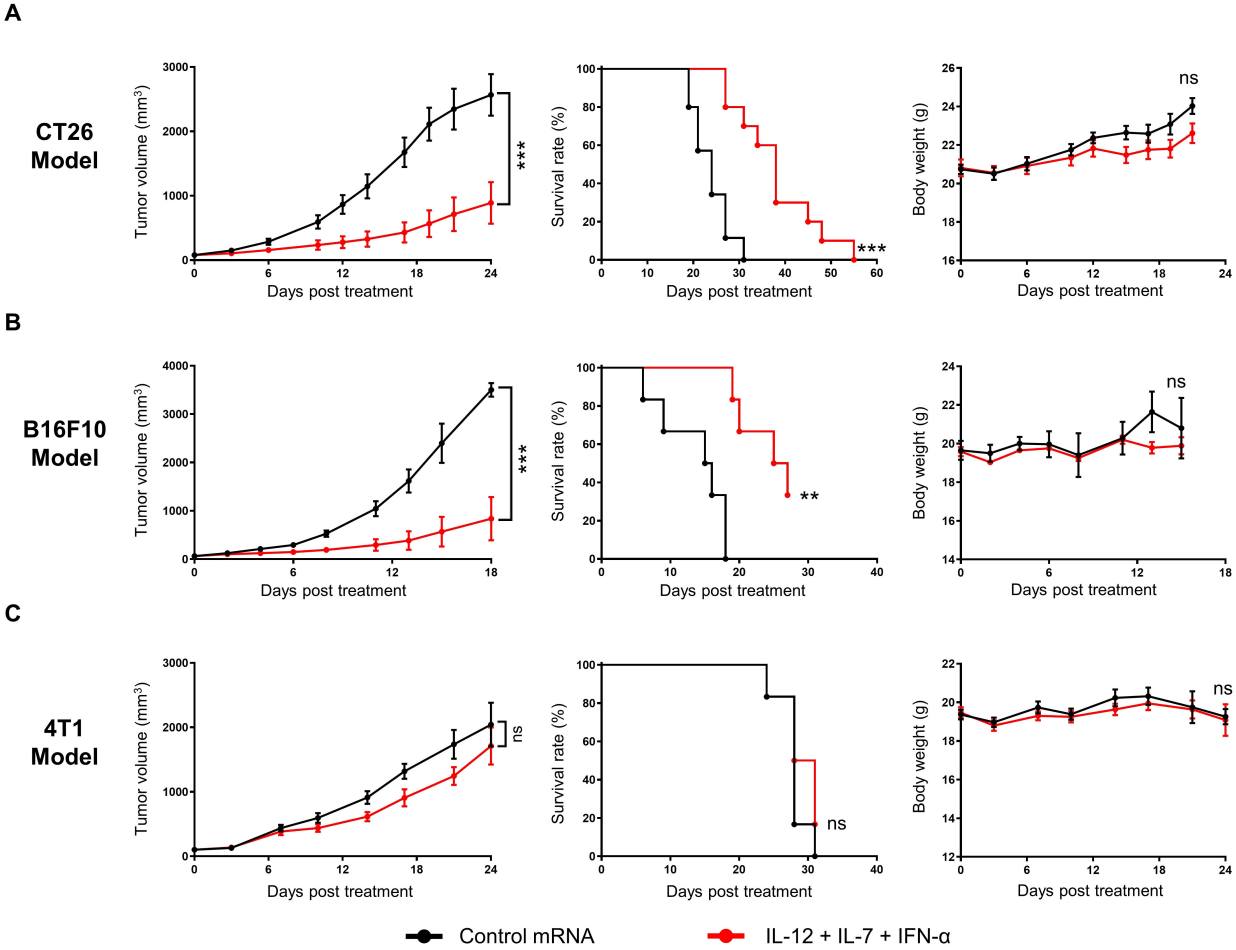
Intratumoral administration of cytokine mRNA triplet inhibited tumor growth and prolonged survival in syngeneic tumor models. **(A–C)** Mice bearing subcutaneous CT26 **(A)**, B16F10 **(B)**, or 4T1 **(C)** tumors were treated with 3 μg of cytokine mRNA triplet (1 μg of each) or 3 μg of luciferase mRNA (control) once a week for 3 consecutive weeks, and their tumor volumes (left) and body weights (right) were determined and Kaplan–Meier survival curves (middle) were generated. Data are presented as the mean ± standard error of the mean, with n = 10 for **(A)** and n = 6 for **(B, C)**. Statistical analysis for tumor volumes and body weights was conducted with two-way ANOVA; ns, not significant, ***P ≤ 0.001. Survival curves were analyzed with a Log-rank (Mantel-Cox) test; ns, not significant, **P ≤ 0.01, ***P ≤ 0.001.

The anti-tumor activity of IL-12/IL-7/IFN-α mRNA triplet was investigated in subcutaneous syngeneic tumor-bearing mice. Repeated intratumoral administration of low-dose cytokine mRNA triplet (3 μg/mouse/injection, 1 μg of each cytokine mRNA) prolonged survival and inhibited tumor growth by 71.04%, 81.01%, and 16.24% [tumor growth inhibition (TGI) index] in CT26 colorectal tumor model (**Figure 3A**), B16F10 melanoma model (**Figure 3B**), and 4T1 breast cancer model (**Figure 3C**), respectively. The cytokine mRNA treatment was well tolerated, with no notable body weight loss or obvious adverse reactions during the experimental period (**Figure 3**).

**Figure 4:**
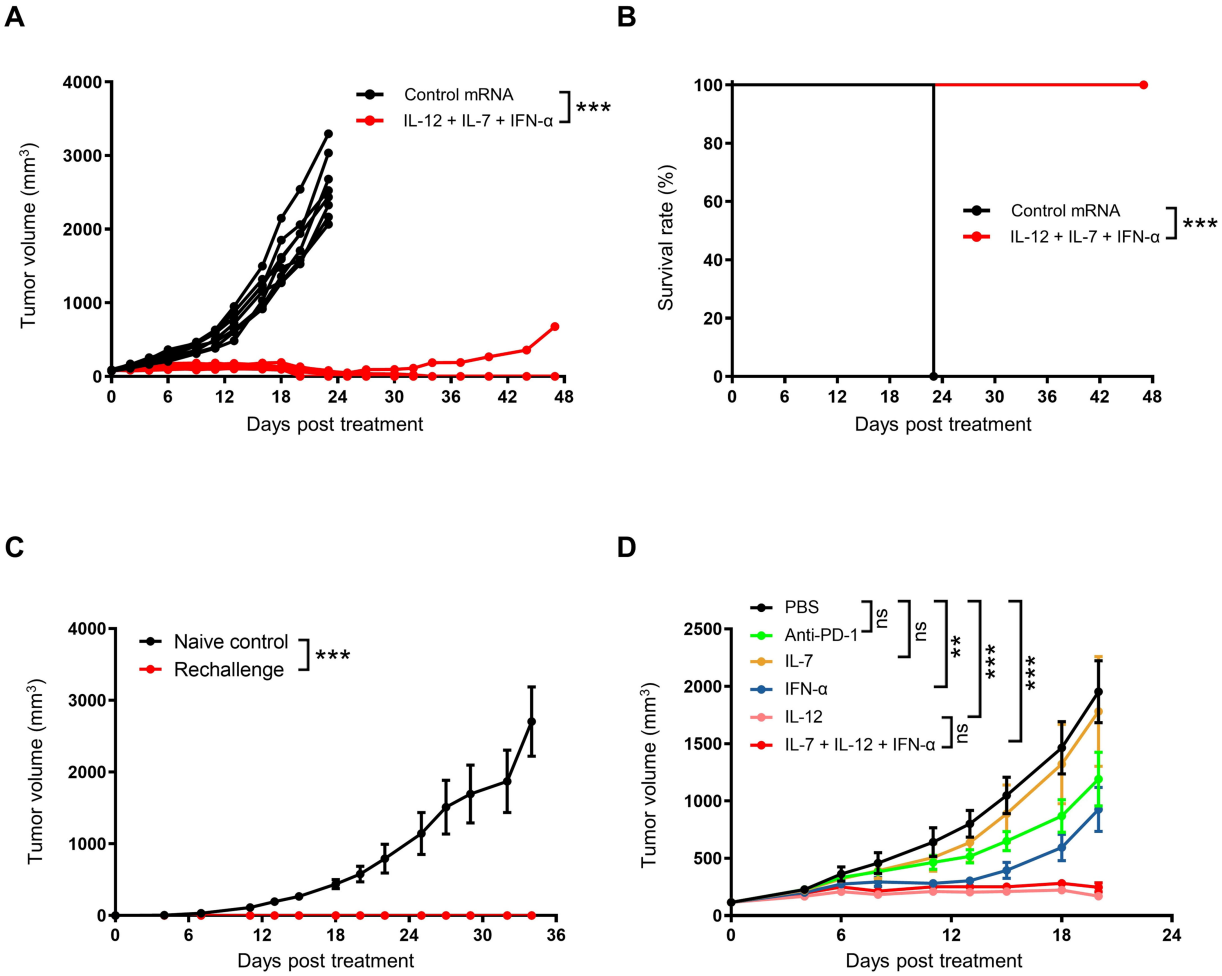
Intratumoral administration of cytokine mRNA triplet induced anti-tumor immune memory. **(A, B)** Subcutaneous CT26 tumor-bearing were treated with 30 μg of cytokine mRNA triplet (10 μg of each) or 30 μg of luciferase mRNA (control) once a week for 3 consecutive weeks, and the individual tumor volumes was shown **(A)** and Kaplan–Meier survival curves **(B)** were generated. n = 8. **(C)** On day 47 after the first treatment, the cytokine mRNA-treated mice were rechallenged with an autologous CT26 tumor, and their tumor volume was determined. Naïve mice were rechallenged as controls. **(D)** CT26 tumor-bearing mice were treated with 30 μg of cytokine mRNA (30 μg of individuals or 10 μg each in a triplet) or equal volume of PBS (control) once a week for 3 consecutive weeks or 10 mg/kg of anti-PD-1 antibodies twice a week for 3 consecutive weeks, and their tumor volumes were determined. Data in **(C, D)** are presented as the mean ± standard error of the mean, with n = 3 (naïve control) and n = 6 (rechallenge and D). Statistical analysis for tumor volumes **(A, C, D)** was conducted through two-way ANOVA with Sidak’s multiple comparison test; ns, not significant, **P ≤ 0.01, ***P ≤ 0.001. Statistical analysis for survival curves **(B)** was conducted with a Log-rank (Mantel-Cox) test; ***P ≤ 0.001.

We further investigated the long-term anti-tumor efficacy of cytokine mRNA triplet. In the CT26 syngeneic model, a high dose of cytokine mRNA triplet (30 μg/mouse/injection, 10 μg for each cytokine mRNA) prolonged survival and led to complete tumor regression in all the treated mice, and 87.5% of mice remained tumor-free for at least 47 d (**Figures 4A, B**). In addition, mice with complete tumor regression of CT26 tumors showed resistance to tumor rechallenge with autologous CT26 tumors at distal sites, while all naïve mice succumbed to significant tumor progression (**Figure 4C**). These results indicate that the cytokine mRNA-treated mice developed anti-tumor immunologic memory.

To further explore the anti-tumor activity of each cytokine, CT26 tumor-bearing mice were treated with individual cytokine mRNAs. Among the three cytokines, IL-12 exhibited the highest efficacy (91.26% TGI at day 20 after the first treatment), followed by IFN-α (53.89% TGI at day 20 after the first treatment) and IL-7 (10.41% TGI at day 20 after the first treatment). The anti-tumor activity of IL-12 was not significantly different from that of the cytokine mRNA triplet (88.2% TGI at day 20 after the first treatment), which may be due to the saturated levels of mRNAs in the high-dose treatment. Notably, cytokine mRNA triplet exhibited superior anti-tumor activity compared with anti-PD-1 therapy (37.2% TGI at day 20 after the first treatment), which is an ICB therapy used as a standard treatment for several solid tumor indications (**Figure 4D**).

### Anti-PD-1 treatment augmented cytokine mRNA-mediated anti-tumor activity

**Figure 5:**
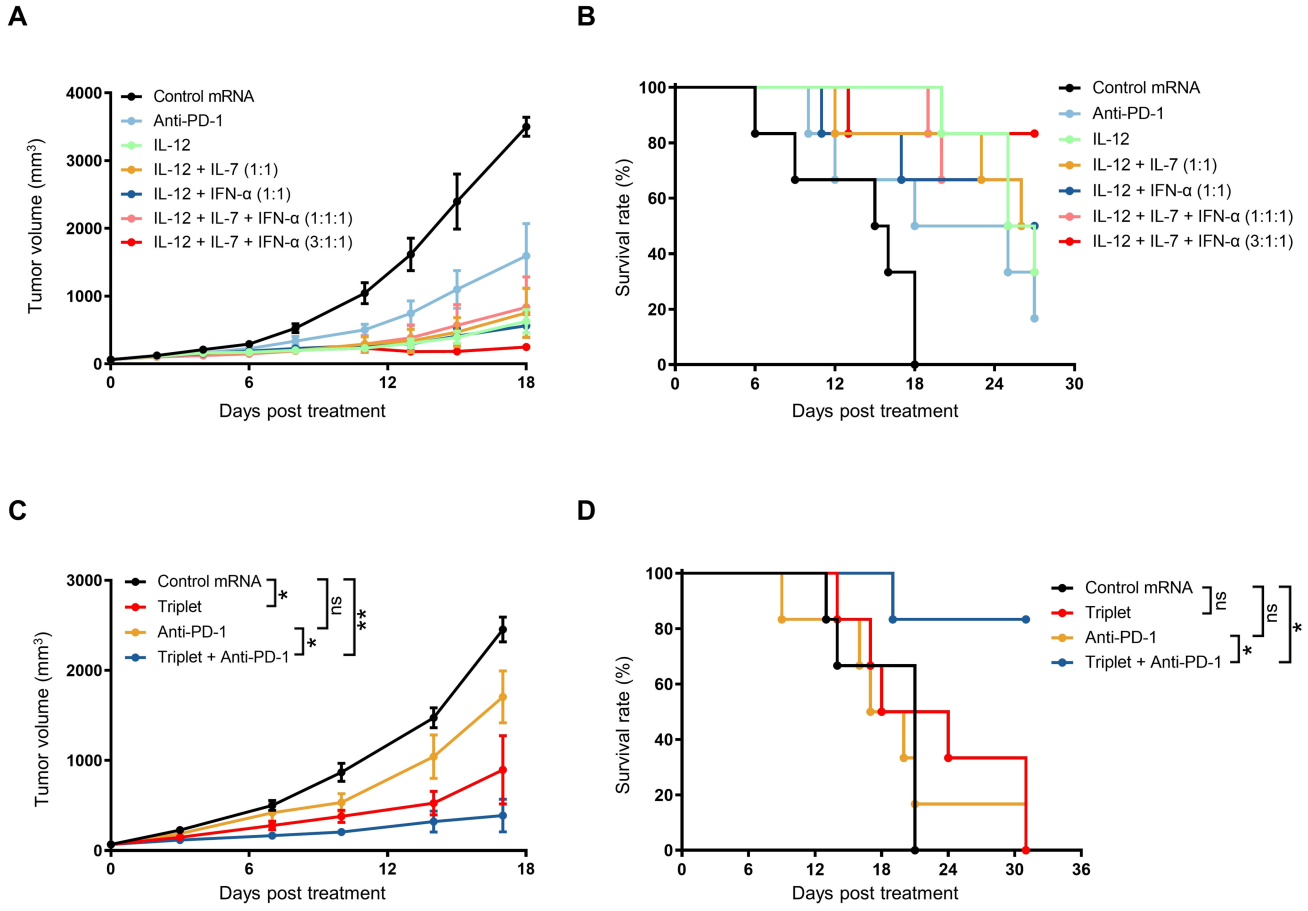
Combination treatment with cytokine mRNA triplet and ICB led to improved anti-tumor efficacy. **(A, B)** Subcutaneous B16F10 tumor-bearing mice were treated with 3 μg of cytokine mRNA (3 μg of individuals; 1.5 μg each of doublets; 1 μg each of triplet; or 1.8 μg of IL-12 mRNA, 0.6 μg of IL-7 mRNA, and 0.6 μg of IFN-α mRNA) or 3 μg of luciferase mRNA (control) once a week for 3 consecutive weeks or 10 mg/kg of anti-PD-1 antibodies twice a week for 3 weeks, and their tumor volumes **(A)** were determined and Kaplan–Meier survival curves **(B)** were generated. **(C, D)** Subcutaneous B16F10 tumor-bearing mice were treated with 0.5 μg of cytokine mRNA triplet (1:1:1 of IL-12/IL-7/IFN-α) or 0.5 μg of luciferase mRNA (control) once a week for 3 consecutive weeks and/or 10 mg/kg of anti-PD-1 antibody twice a week for 3 consecutive weeks, and their tumor volumes **(C)** were determined and Kaplan–Meier survival curves **(D)** were generated. Data are presented as the mean ± standard error of the mean with n = 6. Statistical analysis for tumor volumes **(A, C)** was conducted through two-way ANOVA with Sidak’s multiple comparison test; ns, not significant, *P ≤ 0.05, **P ≤ 0.01. Statistical analysis for survival curves **(B, D)** was conducted with a Log-rank (Mantel-Cox) test; ns, not significant, *P ≤ 0.05, **P ≤ 0.01.

Since IL-12 exhibited the highest efficacy among the three cytokines, we compared the anti-tumor efficacy of IL-12 mRNA and cytokine mRNA combinations (at the same total dose) in subcutaneous B16F10 tumor-bearing mice. The IL-12 mRNA-treated mice exhibited 84.63% TGI at day 18 after the first treatment and a 33.33% survival rate at the end of the study (**Figures 5A, B**). The anti-tumor efficacies of IL-12/IL-7 and IL-12/IFN-α cytokine mRNA doublets and equiquantity triplet (1:1:1 of IL-12/IL-7/IFN-α) were not significantly different from that of the IL-12 mRNA monotherapy, as evidenced by their comparable TGI indexes and median survival times. Notably, cytokine mRNA triplet with 3:1:1 mass ratio of IL-12, IL-7, and IFN-α exhibited superior anti-tumor activity, with 93.84% TGI at day 18 after the first treatment and 80% survival rate at the end of the study (**Figures 5A, B**). These results indicate that IL-12 plays a predominant role in tumor eradication and that IL-7 and IFN-α enhance IL-12-induced anti-tumor activity.

IL-12 treatment has been reported to induce the release of IFN-γ, which triggers PD-L1 expression in both tumor and myeloid cells (39). Considering that PD-1/PD-L1 interaction can suppress anti-tumor immunity (40), we hypothesized that combination treatment with cytokine mRNA triplet and PD-1/PD-L1 blockade would enhance the anti-tumor efficacy. Indeed, combination treatment with a low dose of mRNA triplet and anti-PD-1 antibodies led to 84.86% TGI on day 17 after the first treatment and an 80% survival rate at the end of the study, while treatment with mRNA triplet or anti-PD-1 antibodies alone conferred 62.87% and 29.36% TGI indexes, respectively (**Figures 5C, D**). These results indicate that PD-1/PD-L1 blockade augmented the anti-tumor efficacy of mRNA triplet.

### Human cytokine mRNA treatment induced tumor regression in humanized mouse models

**Figure 6:**
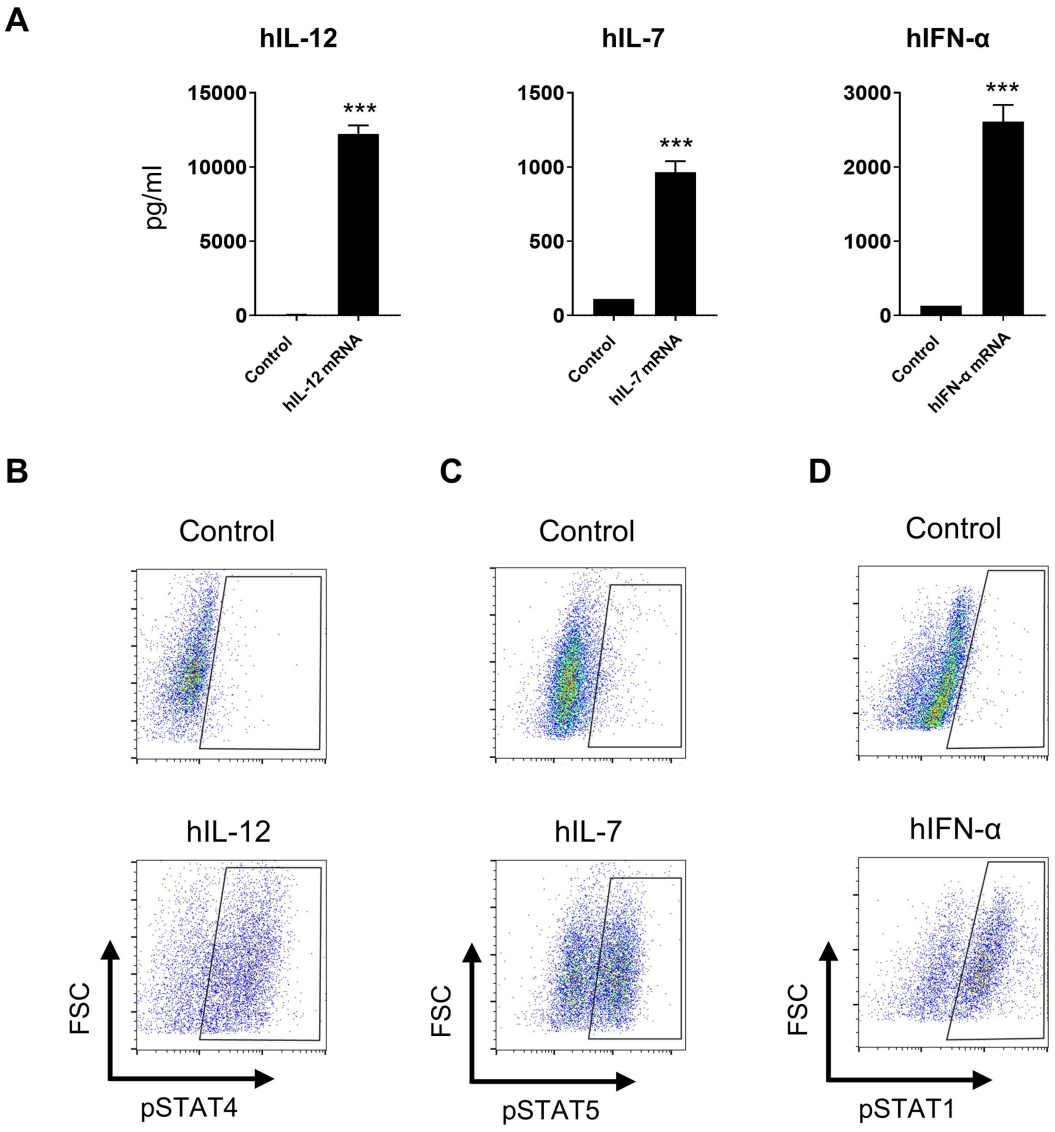
*In vitro* bioactivities of human cytokine mRNAs. **(A)** Cytokine levels in the cultured supernatant of human cytokine mRNA-transfected Jurkat cells. Data are presented as the mean ± standard deviation with n = 3. **(B–D)** Supernatants derived from IL-12, IL-7, and IFN-α mRNA-transfected cells induced STAT4, STAT5, and STAT1 phosphorylation in NK92 cells **(B)**, human PBMCs **(C)**, and THP-1 cells **(D)**, respectively, as indicated by flow cytometry plots. Supernatants derived from luciferase mRNA-transfected cells was used as negative controls. Statistical analysis was conducted using an unpaired, two-tailed Student’s *t*-test; ***P ≤ 0.001. Data are representative of two independent experiments **(A)** or three independent experiments **(B–D)**.

Since the murine cytokine IL-12/IL-7/IFN-α mRNA triplet exhibited significant anti-tumor efficacy in syngeneic mouse models, we generated a human cytokine mRNA triplet for clinical translation. The expression of human cytokine mRNAs was confirmed in the culture supernatant of transfected Jurkat cells (**Figure 6A**), and their bioactivities were demonstrated by phosphor-specific flow cytometry assays. The culture supernatant of IL-12, IL-7, and IFN-α mRNA-transfected Jurkat cells significantly induced STAT4, STAT5, and STAT1 phosphorylation in NK92, PBMCs, and THP-1 cells, respectively (**Figures 6B–D**).

**Figure 7:**
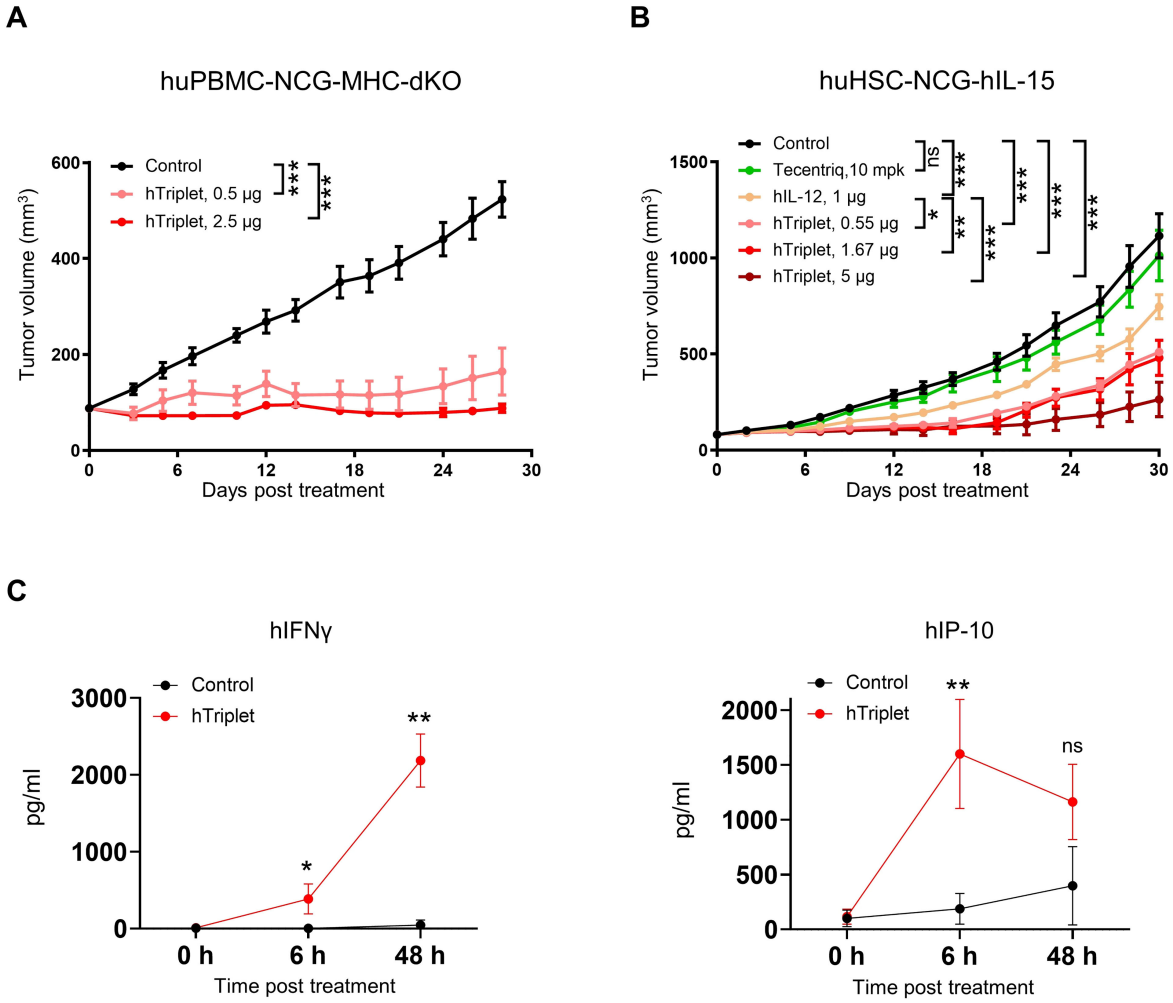
Human cytokine mRNA treatment led to tumor growth inhibition in humanized immune system mouse models. **(A)** NCG-MHC-dKO mice reconstituted with human PBMCs were subcutaneously implanted with MDA-MB-231 cells. Mice with established tumors were intratumorally injected with 0.5 or 2.5 μg of cytokine mRNA triplet (3:1:1 of IL-12/IL-7/IFN-α) or 2.5 μg of luciferase mRNA (control) once a week for 3 consecutive weeks, and their tumor volumes were determined. **(B)** NCG-hIL-15 mice reconstituted with human HSCs were subcutaneously implanted with MDA-MB-231 cells. Mice with established tumors were intratumorally injected with 1 μg of IL-12 mRNA; 0.55, 1.67, or 5 μg of cytokine mRNA triplet (3:1:1 of IL-12/IL-7/IFN-α) or 5 μg of luciferase mRNA (control) once a week for 3 consecutive weeks; or 10 mg/kg of Tecentriq twice a week for 3 weeks, and their tumor volumes were determined. **(C)** The MDA-MB-231 tumor-bearing huHSC-NCG-hIL-15 mice were intratumorally administered with 5 μg cytokine mRNA triplet (3:1:1 of IL-12/IL-7/IFN-α) or 5 μg of luciferase mRNA (control), and their serum IFNγ and IP-10 levels were determined by cytometric bead array analysis 6 and 48 h post-treatment. Tumor volume data are presented as the mean ± standard error of the mean, with n = 6 for **(A)** and 8 for **(B)**. Cytokine concentration data in **(C)** are presented as the mean ± standard deviation with n = 3. Statistical analysis for tumor volumes **(A, B)** was conducted through two-way ANOVA with Sidak’s multiple comparison test; ns, not significant, *P ≤ 0.05, **P ≤ 0.01, ***P ≤ 0.001. Statistical analysis for cytokine production **(C)** was conducted using an unpaired, two-tailed Student’s *t*-test; ns, not significant, *P ≤ 0.05, **P ≤ 0.01.

To explore the *in vivo* anti-tumor activity of human cytokine mRNAs, we established two humanized immune system mouse models, huPBMC-NCG-MHC-dKO and huHSC-NCG-hIL15. The huPBMC-NCG-MHC-dKO mice, which are highly immunodeficient NCG mice with MHC class I and II double knockout to alleviate graft-versus-host disease (GvHD), were engrafted with human PBMCs. Meanwhile, the huHSC-NCG-hIL15 mice, with a knock-in of human IL-15 to maintain the survival and proliferation of human NK cells, were engrafted with huHSCs. Thereafter, MDA-MB-231 cells were subcutaneously implanted in both mouse models. In the huPBMC-NCG-MHC-dKO model, treatment with 0.5 and 2.5 μg of mRNA cytokines significantly improved tumor growth, with 67.83% and 83.33% TGI indexes, respectively, at day 28 after the first treatment (**Figure 7A**). In the huHSC-NCG-hIL15 model, treatment with 0.55, 1.67, and 5 μg cytokine mRNA triplet significantly inhibited tumor growth in a dose-dependent manner, with 53.95%, 57.05%, and 77.56% TGI indexes, respectively, at day 30 after the first treatment (**Figure 7B**). Notably, cytokine mRNA triplet exhibited superior anti-tumor efficacy at all tested doses compared with IL-12 mRNA monotherapy and anti-PD-L1 therapy (**Figure 7B**).

In the huHSC-NCG-hIL15 model, intratumoral injection of cytokine mRNA triplet led to increased serum IFNγ and IP-10 levels (**Figure 7C**). Considering that IFNγ (Th1 cytokine) and IP-10 (Th1 chemokine) are pharmacodynamic response markers for IL-12 therapy, our results suggest that cytokine mRNA triplet promoted Th1-polarized anti-tumor inflammation.

### Cytokine mRNA treatment induced tumor microenvironment inflammation

**Figure 8:**
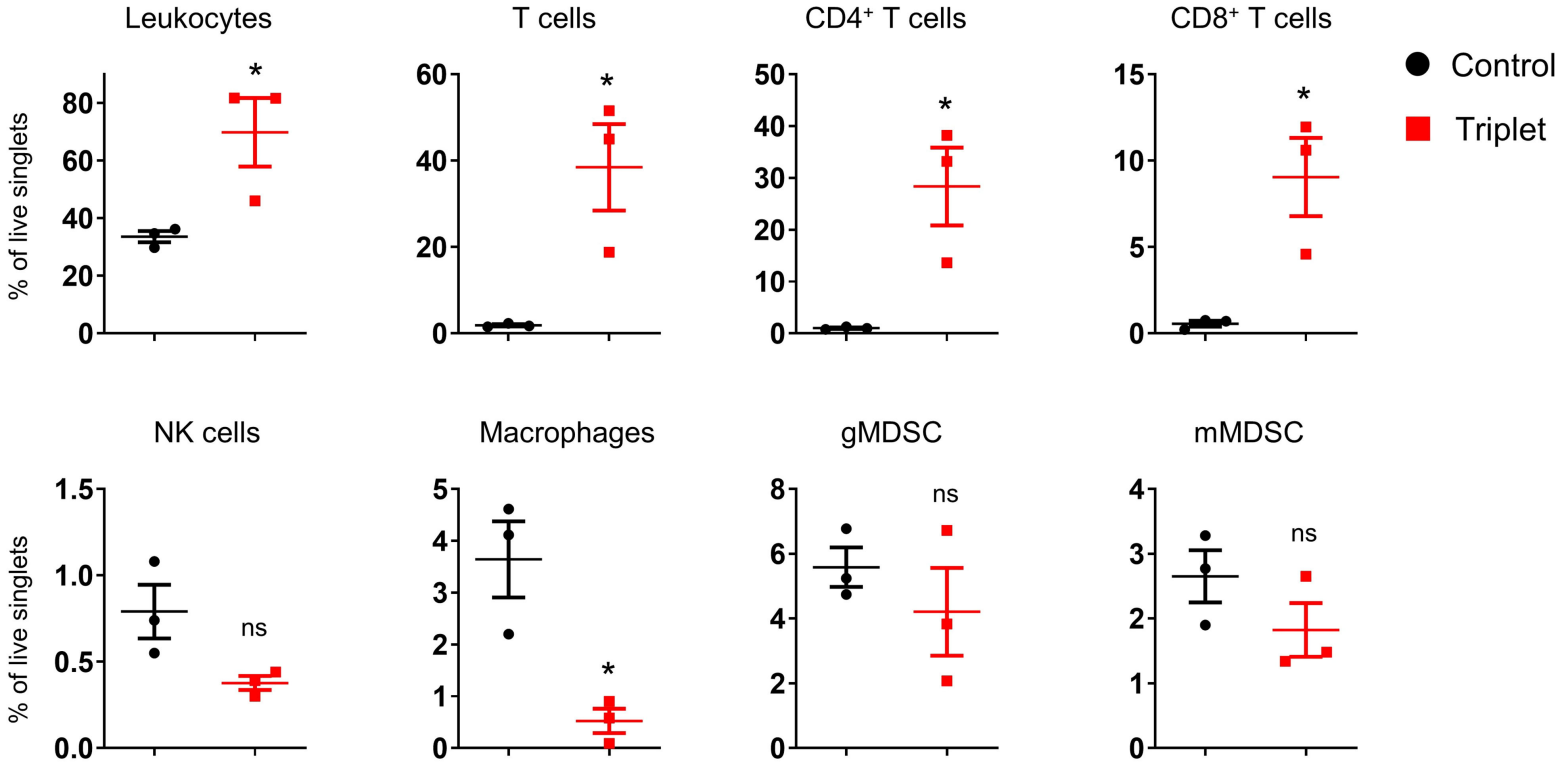
Intratumoral treatment of cytokine mRNAs altered TME in syngeneic tumor model. CT26 tumor-bearing mice were intratumorally injected with 30 μg cytokine mRNA triplet (10 μg of each) or 30 μg of luciferase mRNA (control) for 2 consecutive weeks, and the immune cells that infiltrated into the TME were analyzed by flow cytometry 11 d post-injection. Data are presented as the mean ± standard error of the mean, with n = 3. Statistical analysis was conducted using an unpaired, two-tailed Student’s *t*-test; ns, not significant, *P ≤ 0.05.

We subsequently investigated the *in vivo* immune response to cytokine mRNA triplet in the CT26 tumor-bearing syngeneic mouse model. Intratumoral administration of cytokine mRNA triplet significantly increased leukocyte infiltration, especially CD4^+^ and CD8^+^ T cell infiltration, in the TME (**Figure 8**). These results indicate that cytokine mRNA triplet inhibited tumor growth by triggering inflammation and promoting T cell infiltration in TME.

## Discussion

In this study, we demonstrated the therapeutic potential of cytokine mRNA cocktail encoding IL-12, IL-7, and IFN-α in both syngeneic mouse models and humanized immune system mouse models. Intratumoral administration of cytokine mRNA triplet inhibited *in situ* tumor growth, as well as induced systemic anti-tumor immune memory. Moreover, combination treatment with ICB further enhanced the anti-tumor activity of cytokine mRNA triplet. Furthermore, cytokine mRNA treatment induced inflammation and promoted T cell infiltration in the TME.

Although cytokine mRNA triplet confers anti-tumor efficacy in CT26 colorectal cancer and B16F10 melanoma-bearing syngeneic mouse models, as well as in MDA-MB-231 breast cancer-bearing humanized immune system mouse model, the sensitivity of the cytokine treatment varied among the models. Among the syngeneic models, the 4T1 tumor-bearing mouse model was relatively insensitive to cytokine mRNA triplet therapy, which may be attributed to the lack of T cell and NK cell infiltration in the TME of this model (41). T cells and NK cells are cytotoxic cells that are essential for tumor eradication and are the primary responders to cytokines IL-12, IL-7, and IFN-α. The amount of human T cells is abundant in HSC- and PBMC-reconstituted mice (42), which may explain the potent anti-tumor efficacy of the cytokine mRNAs in these two models. This correlation suggests that “hot” tumors (characterized by high lymphocyte infiltration in the TME) may respond better to cytokine mRNA triplet, which should be taken into consideration during patient recruitment in clinical trials. We used subcutaneous tumor models in this study; however, orthotopic tumor models, recapitulating the TME in patients, might be more relevant for preclinical evaluation.

Among the three cytokine mRNAs, only IL-12 mRNA monotherapy led to complete tumor regression in the syngeneic CT26 mouse model, suggesting the predominant role of IL-12 in anti-tumor immunity. However, the addition of IL-7 and IFN-α mRNA augmented the anti-tumor activity of IL-12 mRNA in the huHSC-NCG-hIL15 mouse model. Consistently, 3:1:1 cytokine mRNA triplet conferred better anti-tumor efficacy, compared with the same total dose of IL-12 singlet, IL-12/IL-7 doublet, or IL-12/IFN-α doublet, in syngeneic B16F10 mouse model. These results suggest the subdominant but also important roles of IL-7 and IFN-α in cytokine mRNA-induced anti-tumor immunity. However, superior anti-tumor immunity was not observed in the syngeneic B16F10 mouse model treated with a 1:1:1 cytokine mRNA triplet, suggesting that the proportion of each cytokine was essential in the mRNA triplet. In the syngeneic CT26 mouse model, the addition of IL-7 and IFN-α mRNA did not further enhance the IL-12 mRNA-induced anti-tumor efficacy, which may be due to the saturated levels of cytokine mRNAs in the high dose (30 μg/mouse/injection). Additionally, cytokine mRNA mixture may balance the immune responses induced by IL-12 mRNA treatment alone.

Intratumoral administration of IL-12, IL-7, and IFN-α cytokine mRNAs induced TME inflammation, characterized by robust T cell infiltration and IFNγ (Th1 cytokine) and IP-10 (Th1 chemokine) secretion. Although previous studies showed that NK cells are essential anti-tumor immune cells that can be activated by IL-12 or IL-12/IL-7 (16, 18), the NK cells were found to be lower in the TME and were slightly downregulated by cytokine mRNA treatment in our study. This may be due to the late detection timepoint, as activated NK cells (but not T cells) were reported to be associated with early cytokine mRNA responses and rapid control of lung lesions in a metastatic lung cancer mouse model (43). In addition, previous studies revealed that NK cells are dispensable for IL-12 mRNA-induced anti-tumor immunity in MC38 tumors (44) and that they played a minimal role in eradicating B16F10 tumors during treatment with IL-12/IL15/IFN-α/GM-CSF mRNA cocktail (43).

For clinical translation, we generated the human cytokine mRNA triplet and demonstrated its anti-tumor efficacy in humanized immune system mouse models. However, these models have several limitations. Neither the HSC model nor the PBMC model can fully recapitulate the human immune system. In the huHSC-IL-15 model, human T cells and NK cells, but not myeloid cells, were well-reconstituted, while in the PBMC model, only T cells were well-reconstituted. Furthermore, GvHD, primarily induced by reconstituted human T cells, is commonly observed in these models, leading to early mortality of mice, which is exacerbated by cytokine mRNA treatment. To expand the therapeutic window, PBMC donors can be pre-screened in mouse models, and those with moderate human T cell reconstitution capacity and minor GvHD reaction can be selected to develop the humanized mouse model. However, pre-screening is infeasible for the HSC model, because the total amount of HSCs from one donor is usually insufficient to reconstitute more than 20 mice.

Immunotherapies with ICB have remarkably changed the paradigm of cancer therapy, and significantly improved clinical outcome for the treatment of malignancy. However, the overall efficacy of ICB monotherapy is relatively low, and many tumors are resistant to ICIs. In this study, we have demonstrated that IL-12/IL-7/IFN-α mRNA triplet induces much higher anti-tumor potency, compared with that induced by anti-PD-1/PD-L1 therapy in both syngeneic and humanized mouse models. Combining ICI further enhanced anti-tumor efficacy of cytokine mRNA triplet. Additionally, compared with systemic immunocytokine-based therapies, local administration of cytokine mRNAs is suggested to limit the toxicities induced by systemic exposure to the drug (24). We have confirmed the intratumoral expression, good tolerability and potent anti-tumor activity of mRNA triplet. Collectively, our data suggest that IL-12/IL-7/IFN-α mRNA triplet as a well-tolerated and efficacious treatment could target unmet medical needs.

In summary, we exploited local immunotherapy of LNP-encapsulated mRNA cocktail encoding cytokines IL-12, IL-7, and IFN-α. Our results highlighted the significant anti-tumor activity of cytokine mRNA triplet in several mouse models and confirmed its mechanism of action.

## Data Availability

The original contributions presented in the study are included in the article/supplementary material. Further inquiries can be directed to the corresponding author.
